# Human conditionally immortalized neural stem cells improve locomotor function after spinal cord injury in the rat

**DOI:** 10.1186/scrt219

**Published:** 2013-06-07

**Authors:** Takashi Amemori, Nataliya Romanyuk, Pavla Jendelova, Vit Herynek, Karolina Turnovcova, Pavel Prochazka, Miroslava Kapcalova, Graham Cocks, Jack Price, Eva Sykova

**Affiliations:** 1 Institute of Experimental Medicine, Academy of Sciences of the Czech Republic, Prague, Czech Republic; 2 Department of Neuroscience, Second Faculty of Medicine, Charles University, Prague, Czech Republic; 3 MR-Unit, Department of Diagnostic and Interventional Radiology, Institute for Clinical and Experimental Medicine, Prague, Czech Republic; 4 Institute of Psychiatry, King’s College London, London, UK

**Keywords:** Human fetal neural stem cells, spinal cord injury, motor neuron differentiation, trophic support, neuroregeneration

## Abstract

**Introduction:**

A growing number of studies have highlighted the potential of stem cell and more-differentiated neural cell transplantation as intriguing therapeutic approaches for neural repair after spinal cord injury (SCI).

**Methods:**

A conditionally immortalized neural stem cell line derived from human fetal spinal cord tissue (SPC-01) was used to treat a balloon-induced SCI. SPC-01 cells were implanted into the lesion 1 week after SCI. To determine the feasibility of tracking transplanted stem cells, a portion of the SPC-01 cells was labeled with poly-L-lysine-coated superparamagnetic iron-oxide nanoparticles, and the animals grafted with labeled cells underwent magnetic resonance imaging. Functional recovery was evaluated by using the BBB and plantar tests, and lesion morphology, endogenous axonal sprouting and graft survival, and differentiation were analyzed. Quantitative polymerase chain reaction (qPCR) was used to evaluate the effect of transplanted SPC-01 cells on endogenous regenerative processes.

**Results:**

Transplanted animals displayed significant motor and sensory improvement 2 months after SCI, when the cells robustly survived in the lesion and partially filled the lesion cavity. qPCR revealed the increased expression of rat and human neurotrophin and motor neuron genes. The grafted cells were immunohistologically positive for glial fibrillary acidic protein (GFAP); however, we found 25% of the cells to be positive for Nkx6.1, an early motor neuron marker. Spared white matter and the robust sprouting of growth-associated protein 43 (GAP43)^+^ axons were found in the host tissue. Four months after SCI, the grafted cells matured into Islet2^+^ and choline acetyltransferase (ChAT)^+^ neurons, and the graft was grown through with endogenous neurons. Grafted cells labeled with poly-L-lysine-coated superparamagnetic nanoparticles before transplantation were detected in the lesion on T_2_-weighted images as hypointense spots that correlated with histologic staining for iron and the human mitochondrial marker MTCO2.

**Conclusions:**

The transplantation of SPC-01 cells produced significant early functional improvement after SCI, suggesting an early neurotrophic action associated with long-term restoration of the host tissue, making the cells a promising candidate for future cell therapy in patients with SCI.

## Introduction

Spinal cord injury (SCI) remains a very complex medical and psychological challenge, both for patients and their relatives and for the involved physicians. The unprecedented success of SCI research in the past few years has resulted in significant advances [1,2]; nevertheless, clinical treatment is still limited to the reduction of pain and swelling and the prevention of secondary injury by the administration of antiinflammatory drugs. Pathologic changes after SCI are complex and include the interruption of ascending and descending pathways, the loss of neurons and glial cells, inflammation, scar formation, and demyelination [3,4]. These events suggest a number of different steps required for SCI repair, such as minimizing progressive cell death and blocking scar formation; replacing lost cells and stimulating the injured cord to produce new cells; reconnecting injured nerve fibers with their original targets or with substitute targets; and maximizing the function of the spared nerve fibers by repairing their myelin sheaths. Because of their specific properties, stem cells may eventually play a role in many or all of these processes. Therefore, cell-transplantation therapies have become a major focus in preclinical research as a promising strategy for the treatment of SCI [5].

Among the many different types of stem cells available today, neural stem and progenitor cells (NSCs) are particularly useful tools for transplantation therapy, because they have the ability to provide an unlimited source of neurons, oligodendrocytes, and astrocytes for the treatment of neurologic and/or neurodegenerative disorders via cell replacement [6]. NSCs can be isolated from the developing or adult central nervous system and can be safely expanded in chemically defined culture media for an extended period. They have immunomodulatory properties [7] and also are able to produce a number of growth factors that have strong neurotrophic and neuroregenerative effects [8]. In addition, NSCs are more kindred to nervous tissue in comparison to other stem cell types (for example, mesenchymal stem cells [9-11] and olfactory ensheathing cells [12]). A positive effect of transplanted NSCs on functional outcome after SCI was shown in several experiments using animal models [13-16]. Moreover, from a clinical perspective, it is important that implanted human NSCs have been shown to give rise to mature neurons and oligodendrocytes and that they have promoted functional recovery not only in SCI models in small animals, but also in injured dogs [17].

The current study addresses a previously unexplored issue in stem cell transplantation research for spinal cord repair in what are, to our knowledge, the first SCI experiments using a conditionally immortalized line of human fetal neural stem cells derived from the spinal cord (SPC-01). For establishing this line, *c*-mycER^TAM^ technology was used in which a fusion protein comprising a growth-promoting gene, *c-myc*, and a hormone receptor that is regulated by a synthetic drug, 4-hydroxy-tamoxifen (4-OHT), were used to achieve conditional growth control. Because of these manipulations, the immortalizing gene is downregulated on transplantation into the host spinal cord [18]. First, we report that SPC-01 cells survive, engraft, differentiate and communicate with the host environment after traumatic SCI in rats. Second, we demonstrated that transplanted SPC-01 cells survive in the lesion site while being tracked by *in vivo* MRI by using poly-L-lysine-coated superparamagnetic iron-oxide nanoparticles. Third, we showed that the transplantation of SPC-01 cells into the lesioned rat spinal cord improves functional outcome by partially bridging the spinal cord lesion and providing trophic support to the spared axons in the injured tissue.

## Methods

### Human fetal neural stem cells SPC-01

The human spinal cord cell line (SPC-01) was generated from 10-week-old human fetal spinal cord. Fetal tissue was obtained from Advanced Bioscience Resources (Alameda, CA, USA) after normal terminations and in accordance with nationally (UK and/or USA) approved ethical and legal guidelines [19,20]. Cells were prepared by mechanical and enzymatic dissociation of the fetal spinal cord cervical region into a single-cell suspension. Subsequently, cells were conditionally immortalized with the *c*-mycER^TAM^ construct. This construct generates a *c*-myc protein fused to a mutated estrogen receptor and is transcriptionally active only in the presence of the synthetic ligand 4-hydroxy tamoxifen. The method used is the same as that published in Pollock *et al.*[18]. For easier *in vivo* detection, the SPC-01 cells were transduced with green fluorescent protein (GFP). The GFP-expressing SPC-01 cells were generated by using a lentiviral vector containing a ubiquitous chromatin opening element (UCOE) to prevent silencing on engraftment, as previously described [21]. Transduced SPC-01_GFP^+^ cells were frozen, stored in liquid nitrogen, and used throughout the whole study.

SPC-01-GFP^+^ cells were routinely cultured in tissue-culture flasks freshly coated with laminin (Sigma, St. Louis, MO, USA; 20 μg/ml in DMEM:F12) for 1 hour at 37°C. Growth media comprising DMEM:F12 supplemented with HSA (0.03%) (Baxter Healthcare Ltd., Norfolk, UK); L-glutamine (2 m*M*), human transferrin (100 μg/ml), putrescine dihydrochloride (16.2 μg/ml), human insulin (5 μg/ml), progesterone (60 ng/ml), sodium selenite (selenium) (40 ng/ml), EGF (20 ng/ml), and 4-hydroxy-tamoxifen (4-OHT) (100 n*M*) all from Sigma; FGF (10 ng/ml) (PeproTech, London, UK) was changed 3 times per week. When 70% to 90% confluent, the cells were passaged by using 0.25% TrypZean (Lonza, Basel, Switzerland) for 2 minutes at 37°C followed by 0.25 mg/ml soybean trypsin inhibitor. SPC-01-GFP^+^ cells from passages 26 through 29 were used in all experiments.

For magnetic labeling, the cells were incubated in a culture medium containing 15.4 μg/ml of iron in the form of poly-L-lysine-coated superparamagnetic-iron-oxide (PLL-SPIO) nanoparticles for 72 hours before transplantation [22]. The nanoparticles were washed out by using HBSS (Invitrogen, Paisley, Scotland, UK), and then the cells were harvested as described earlier.

### Animals

Ten-week-old male Wistar rats were obtained, and their body weight ranged between 270 g and 300 g to minimize differences in body size to achieve standardized spinal cord lesions. To complete this study, 83 animals were used. The numbers of animals used for all parts of the study are summarized in Additional file 1: Table S1. All experiments were performed in accordance with the European Communities Council Directive of 24 November 1986 (86/609/EEC) regarding the use of animals in research and were approved by the Ethics Committee of the Institute of Experimental Medicine ASCR, Prague, Czech Republic.

### Spinal cord injury

A balloon-compression lesion was performed in a total of 79 male Wistar rats, as described by Urdzikova *et al*. [11]. In brief, the animals were anesthetized with 2% isoflurane (Forane; Abbott Laboratories, Queenborough, UK) and shaved on the back from C7 to Th1. Under sterile conditions, the skin was cut in the midline from Th7 to Th12. The soft tissue was removed, as well as the spinous processes of vertebrae Th8 to Th11. A 2F Fogarty catheter was inserted into the epidural space and advanced cranially for 1 cm, so that the center of the balloon rested at the Th8 to Th9 level of the spinal cord. The balloon was rapidly inflated with 15 μl of saline for 5 minutes. The catheter was then deflated and removed. The soft tissue and skin were sutured with unresorbable thread, and the animals were allowed to feed and drink *ad libitum*. During the surgical procedure, the body temperature of the animal was maintained at 37°C with a heating pad, and 3% uurane in air was administered at a flow rate of 0.3 L/min. After being returned to their cages, the rats were assisted in feeding and urination until they had recovered sufficiently to perform these functions on their own. The animals received gentamicin sulfate (5 mg/kg) for 3 days to prevent postoperative infections.

### Transplantation

The animals were transplanted 1 week after SCI. This time point is generally accepted as a suitable therapeutic window, as the inflammatory reaction (creating a hostile environment for cell-transplant survival) decreases during the first 7 days, and the glial scar that prevents graft-host tissue communication is not yet developed [23]. The animals were secured in a stereotaxic apparatus with a rat-specific vertebra holder (Cunningham spinal adaptor; Stoelting Co., Wood Dale, IL, USA). The spinal cord was exposed at T8. In total, 5 × 10^5^ SPC-01 cells/5 μl (either unlabeled or labeled with nanoparticles) were injected through a glass pipette into the center of the lesion at a depth of 1 mm below the dorsal surface at a rate of 1 μl/min by using a Nano-Injector (Stoelting). Cells were harvested as described earlier, and the cell suspension was prepared just before the transplantation procedure. The number of cells was determined based on the results obtained from our pilot study, in which 1 × 10^5^ SPC-01 cells/1 μl were also injected into the proximal, central, and distal parts of the lesioned spinal cord. However, the survival rate of the transplanted cells was worse than when injecting 5 × 10^5^ SPC-01 cells/5 μl into the lesion. The glass pipette was kept in place after injection for a further 5 minutes to prevent leakage of the cell suspension. The control group received 5 μl of saline. Triple-drug immunosuppression was used to prevent graft rejection [24]. Cyclosporine A (10 mg/kg), azathioprine sodium (2 mg/kg), methylprednisolone (2 mg/kg, tapered to 0.5 mg/kg), and ampicillin (50 to 100 mg/kg) were administered 1 day before transplantation and throughout the experiment (for 2 months or 4 months).

### MRI

To visualize the grafted cells *in vivo*, part of the cells were labeled with poly-L-lysine-coated superparamagnetic iron-oxide (PLL-SPIO) nanoparticles, and four animals grafted with labeled cells, and four animals injected with saline underwent magnetic resonance imaging (MRI). MR images were taken 5 days after SCI (that is, before transplantation), and 1, 4, and 8 weeks after transplantation (that is, 2, 5, and 9 weeks after SCI). In total, 5 × 10^5^ SPC-01 cells labeled with PLL-SPIO nanoparticles were injected in the same way as described earlier.

MR images were obtained with a 4.7-T spectrometer (Bruker BioSpin, Ettlingen, Germany) by using a homemade surface coil incorporated in an animal holder dedicated for spinal cord measurements. The rats were anesthetized by 1.5% to 2% isoflurane in air. The respiration of the animals was monitored during the MR measurements. The rats were examined on their backs. After initial pilot scans, which were used for proper geometry setting, a turbo-spin echo sequence was used for the acquisition of five slices in a sagittal orientation. The sequence parameters were turbo factor, 16; repetition time, TR = 2,000 ms; effective echo time, TE = 69.9 msec; number of acquisitions, AC = 48; field of view, FOV = 5 × 3 cm; matrix size, MTX = 256 × 256; slice thickness, 0.75 mm.

### Functional analysis

For motor testing, hindlimb locomotor activity after SCI was assessed with the Basso, Beattie, and Bresnahan (BBB) test [25] (SCI and SPC-01, *n* = 20; SCI only, *n* = 16). The rats were placed on a floor within a circular enclosure. Their hindlimb joint movement, paw placement, weight support, forelimb-hindlimb coordination, and so on, were evaluated by using a 0 to 21-point scale.

For sensitivity testing, hindpaw-withdrawal latency to noxious thermal stimuli was assessed with a Plantar Test apparatus (Ugo Basile, Comerio, Italy) (the same animals that underwent BBB testing). The animals were placed in a clear plastic chamber and acclimated for 10 minutes until becoming quiescent. The hindpaw received a heat stimulus through a glass plate. The withdrawal latencies were measured 5 times for each hindpaw at 5-minute intervals.

The motor function of the hindlimbs was also examined with the walking-beam test (SCI and SPC-01, *n* = 8; SCI only, *n* = 8). The apparatus consisted of a 3.4-cm-wide and 140-cm-long wooden rectangular beam. A goal box was placed at one end. The central 1 m of the beam was used to evaluate the walking distance. The latency and the trajectory to traverse the beam were recorded with a video-tracking system (TSE-Systems Inc., Bad Homburg, Germany) for a maximum of 60 seconds. After pretraining, two trials were given each day for 3 consecutive days. The animals were examined before surgery and every week from the second week after the injection of SPC-01 cells or saline. A 0 to 7-point scale modified from Goldstein [26] was used to evaluate the locomotor function.

The average of five values was used for statistical evaluation. The data are expressed as mean ± SEM. All data were compared between the sham-operated group and the transplanted group by a two-sample *t* test for independent samples, if the two samples had equal variances. If they had unequal variances, the Mann–Whitney test was used for evaluation. A *P* value <0.05 was considered statistically significant. All behavioral tests were performed by two independent blind observers.

### Histologic and immunohistochemical analysis

To analyze the volume of the spared white and gray matter and the extent of axonal sprouting, animals with SCI only (*n* = 8) and animals grafted with SPC-01 cells (*n* = 7) were killed 8 weeks after transplantation by transcardial perfusion. For perfusion, the animals were deeply anesthetized with ketamine (100 mg/kg) and xylazine (20 mg/kg). Their chests were opened, and transcardial perfusion was performed with phosphate buffer until the liver tissue was clean, followed by perfusion with a 4% paraformaldehyde (PFA) solution in phosphate buffer. A 2-cm-long segment of the spinal cord was dissected between 1 cm cranial and 1 cm caudal to the injury epicenter. Serial cross sections, 5 μm thick, were cut by using a K400 microtome (Microm GmbH, Walldorf Germany) after paraffin embedding, and were stained with either Luxol-fast blue (1 g of Luxol-fast blue dissolved in 100 ml of 96% ethanol with 5 ml of 10% acetic acid) or cresyl violet (0.25 g of cresyl violet dissolved in 100 ml of distilled water with 1 ml of 10% acetic acid) to distinguish the white and gray matter or by an anti-GAP43 antibody (Millipore, Billerica, MA, USA; MAB347) to evaluate axonal sprouting. For volumetric measurements, six sections were selected at 1-mm intervals along the rostrocaudal axis, and whole images of the spinal cord were taken with an Axioskop 2 plus microscope (Carl Zeiss AG, Oberkochen, Germany) and analyzed with ImageJ software (Wayne Rasband, National Institutes of Health, USA) [11]. Axonal sprouting was expressed as the mean number of GAP43-positive fibers per section [27]. High-magnification images of transverse sections, separated by a 1-mm distance in all animal groups stained for GAP43, were taken, and GAP43-positive fibers were manually counted. Each counted fiber was marked with red color to avoid double counting.

For immunohistochemical evaluation of the grafts, animals grafted with SPC-01 cells were killed 8 (*n* = 14) or 17 weeks (*n* = 5) after transplantation by transcardial perfusion. To monitor the survival, migration, and differentiation of the transplanted cells, serial longitudinal sections of the spinal cord (14 μm) were cut through the areas of interest, by using a Leica CM1850 cryostat (Leica Mikrosysteme GmbH, Vienna, Austria). To identify human stem cells transplanted into the rat spinal cord, antibodies directed against human nuclei (HuNu) (Chemicon, Temecula, CA, USA) and human mitochondrial marker (MTC02) (Abcam, Cambridge, UK) were used. To follow the fate of the transplanted stem cells and their communication with the host tissue, antibodies directed against nestin, neurospecific enolase (NSE), chondroitin sulfate proteoglycan (NG2), calcitonin gene-related peptide (CGRP) (all Millipore); neurofilament 160 (NF160), neurofilament 200 (NF200), glial fibrillary acidic protein (GFAP) (all Sigma, St. Louis, MO, USA); Olig2, choline acetyltransferase (ChAT), 2ʹ,3ʹ-cyclic nucleotide 3ʹ-phosphodiesterase (CNPase), Ki67 (all Abcam); Islet2, Nkx6.1 (DSHB, Iowa City, IA, USA), and Tau (Dako Cytomation, Glostrup, Denmark) were used. To visualize primary antibody reactivity, appropriate secondary antibodies were used: goat anti-mouse IgG conjugated with Alexa-Fluor 488 and 594 and goat anti-rabbit IgG conjugated with Alexa-Fluor 594 (Molecular Probes, Eugene, OR, USA). Confocal images were taken with a Zeiss LSM 5 Duo confocal microscope (Carl Zeiss).

To count the number of transplanted cells, every sixth longitudinal cryostat section (14-μm thickness) of the spinal cord was chosen for imaging the whole graft area. Images were taken with an Observer D1 microscope (Carl Zeiss). The surviving human cells, which were recognized as HuNu-positive cells, were counted in each section by using ImageJ software. The total number of cells was estimated according to the volume of the section in which the cells were found. The percentage of surviving transplanted cells was calculated by dividing the estimated total number of surviving cells by the total number of transplanted cells (5 × 10^5^). The percentage of transplanted cells positive for specific markers was calculated as the ratio of Ki-67- or Nkx6.1-positive cells to the total of surviving HuNu-positive cells.

To visualize nanoparticle internalization in rats transplanted with magnetically labeled cells (*n* = 4), potassium ferrocyanide (1 g of K_4_[Fe(CN)_6_]3H_2_O dissolved in 100 ml 0.5% HCl) was applied for 30 minutes to produce ferric ferrocyanide [prussian blue staining for ferric (3^+^) iron].

### RNA extraction and quantitative PCR analysis of gene expression

Total RNA was extracted from rat spinal cord tissue and human fetal neural stem cells by using the RNeasy Lipid Tissue Mini Kit (Cat. no. 74804) and RNeasy Plus Mini Kit (Cat. no. 74134), both from Qiagen GmbH (Hilden, Germany) according to the manufacturer’s instructions. Spinal cord tissue was dissected from animals of three different groups: intact healthy animals without SCI (*n* = 4), animals with SCI only (*n* = 4), and animals grafted with SPC-01 cells (*n* = 4) and killed 8 weeks after transplantation.

The expressions of rat (*Rattus norvegicus*) target genes *Bdnf*, *Vegfa*, *Ngf,* and *Nt3* (*Sort1*) and human (*Homo sapiens*) target genes *BDNF*, *VEGFA*, *NGF*, *NT3* (*SORT1*), *NKX6-1*, *ISL2*, *HB9* (*MNX1*), *SYP,* and *CHAT* were determined by quantitative real-time reverse transcription polymerase chain reaction (qPCR) in a 7500 Real Time PCR System (Applied Biosystems, Foster City, CA, USA) by using TaqMan Gene Expression Master Mix (catalog number 392938) and TaqMan Gene Expression Assays 4331182 (Rn02531967_s1/Bdnf/, Rn01511601_m1/Vegfa/, Rn01533872_m1/Ngf/, Rn01521847_m1/Sort1/, Hs01010223_m1/BDNF-AS1/, Hs00900055_m1/VEGFA/, Hs00171458_m1/NGF/, Hs00361760_m1/SORT1/, Hs00232355_m1/NKX6-1/, Hs00377575_m1/ISL2/, Hs00907365_m1/MNX1/, Hs00300531_m1/SYP/, Hs00252848_m1/CHAT). The qPCR was carried out in a final volume of 20 μl containing 500 ng of extracted RNA. The following thermal profile was used: a single cycle of reverse transcription for 30 minutes at 50°C and 15 minutes at 95°C for reverse transcriptase inactivation and DNA polymerase activation, followed by 40 cycles of denaturation at 95°C for 15 seconds and annealing and extension at 60°C for 1 minute. The results were analyzed by using the integrated 7500 System SDS Software (version 1.3.1). Each data set was normalized with an appropriate TaqMan endogenous control selected by NormFinder [28]. As endogenous control genes, *Actb* (Rn00667869_m1) and *GAPDH* (Hs99999905_m1) were chosen for rat and human target genes, respectively. All qPCR reagents were provided by Applied Biosystems Foster City, CA, USA. Finally, the data were recalculated to relative quantities and transformed to a log2 scale by using the Relative Expression Software Tool (REST) [29]. In the case of the unexpressed *NGF* gene in SPC-01 cells before differentiation, the undetermined cycle of quantification (Cq) value was set to a maximum (for example, 40) for the calculation of the relative expression ratio. All numeric data were presented as mean ± standard deviation and analyzed statistically by using REST.

## Results

### FACS analysis of SPC-01 cells

To characterize the expression of pluripotent and neural markers in human fetal neural stem cells from the SPC-01 line, a series of FACS analyses was performed before transplantation (a description of the FACS analysis method, the results, and figure S1 are presented in the Additional files 2 and 3). The flow-cytometry results revealed that SPC-01 cells display a clear neural profile in terms of their marker expression. At the same time, these cells are negative for pluripotent markers, which suggests that they are safe for use in transplantation experiments.

### BBB test

**Figure 1:**
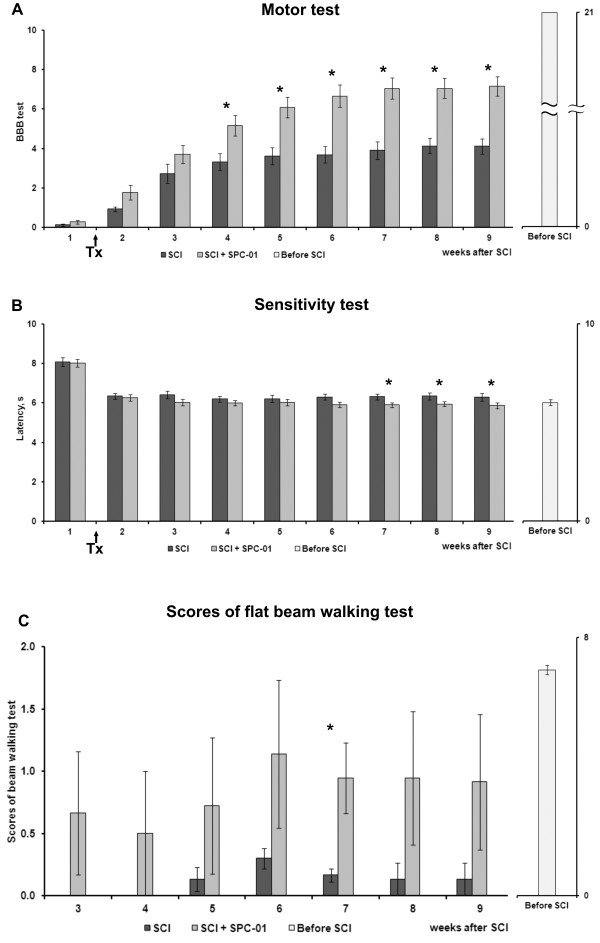
**Functional recovery after SPC-01 cell transplantation.** Hindlimb locomotor function was assessed weekly with the open-field BBB test (**A**). A significant difference between the control and the transplanted animals was found at 4 weeks after SCI (*P < 0.05). The withdrawal latency of the hindpaw in response to a heat stimulus was measured weekly with the Plantar test (**B**). A significant difference in withdrawal latency compared with the controls appeared from 7 weeks after SCI (*P < 0.05). A statistically significant difference between the control and transplanted groups in the scores of the flat beam walking test (**C**) appeared at 7 weeks after SCI (6 weeks after transplantation) (*P < 0.05). Tx marks the day of transplantation.

The motor recovery of the hind legs after SCI was examined weekly by using the BBB test (Figure 1A). The hindlimb function was severely damaged by the balloon compression: BBB scores were between 0 and 2 a week after surgery. The control animals with a balloon-compressed thoracic spinal cord, and saline injection showed spontaneous functional recovery as quickly as the transplanted animals during the first 3 weeks after lesioning. The final score of control animals on the BBB test was 4.19 ± 0.38 at 9 weeks after SCI. In contrast, the transplanted animals started to show a significant improvement of their hindlimb locomotion 4 weeks after SCI (3 weeks after transplantation) compared with the controls (*P* < 0.05) and reached a final BBB score of 6.90 ± 0.48. Extensive joint movement, which is recognized as a flexion angle of more than half of the possible range of motion of the joint, was observed in all joints of the hind legs (three joints in each leg) in 65% of the transplanted animals at the end of the experiments, but in only one animal (6.3%) among the control group. In addition, in transplanted animals, body weight was supported on their hind legs, and paw stepping was seen in 15% of them.

### Plantar test

For sensitivity testing, the withdrawal latency of the hindpaws from a thermal stimulus was examined weekly after SCI (Figure 1B). The average latency determined from five repeated measurements for each paw was 8.08 ± 0.22 seconds in the control animals and 8.13 ± 0.20 seconds in the transplanted animals 1 week after SCI, which decreased to 6.34 ± 0.14 and 6.26 ± 0.18 seconds, 2 weeks after SCI (1 week after transplantation), respectively. The control animals did not improve their latency further; their final average latency at 9 weeks after SCI was 6.30 ± 0.19 seconds. In contrast, the transplanted animals shortened their latency during each subsequent week and started to show significant differences compared with the controls at 7 weeks after SCI (*P* < 0.05).

### Beam-walking test

The ability to traverse a beam with a flat surface was examined weekly. Rats with SCI, injected with saline, were not able to transverse the beam at all, because no signs of weight support and/or stepping were observed (Figure 1C). However, after SPC-01 treatment, the rats started to maintain their body weight on the beam for 60 seconds, and some of them could traverse the beam. A statistically significant difference appeared at 7 weeks after SCI (6 weeks after transplantation) between the control (0.2 ± 0.06) and transplanted (2.3 ± 0.33) groups (*P* < 0.05).

### *In vivo* imaging

To check the feasibility of tracking transplanted stem cells, four animals were grafted with SPC-01 cells labeled with PLL-SPIO nanoparticles. The cells were labeled before transplantation. The viability of the labeled cells was 88%, and the labeling efficiency was 57%, which means that 57% of cells were labeled. (The methods used to evaluate cell viability and labeling efficiency are described in Additional file 2).

**Figure 2:**
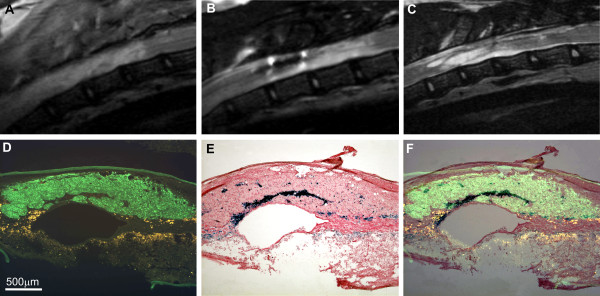
**T**_**2**_**-weighted MR images of the injured spinal cord before and after SPC-01 cell transplantation.** T_2_-weighted MR images of a spinal cord lesion 5 days after lesion induction, before transplantation (**A**), a spinal cord with a cell graft 8 weeks after cell transplantation (**B**), and a control spinal cord lesion 8 weeks after saline injection (**C**). Two serial sections were stained for MTC02 (**D**) and iron (**E**) and their overlay (**F**).

The spinal cord lesion was visible on T_2_-weighted MR images 5 days after lesioning as a hyperintense signal (Figure 2A), probably representing edema. The grafted cells were detected in the lesion on T_2_-weighted images as a strong hypointense area (Figure 2B) in the cranial part of the lesion, compared with the hyperintense signal in the nontransplanted lesioned spinal cord (Figure 2C). These MR images corresponded to the MTCO2- and Prussian blue-stained tissue sections (Figure 2D, E). The grafted SPC-01 cells were identified as a packed cell mass. However, the Prussian blue-stained nanoparticles were more accumulated inside the cell mass compared with the margins of the immunostained cell mass, indicating that some of the cells expelled the label (Figure 2F). We speculate that the expulsion of nanoparticles can happen because of the differentiation of stem cells, particularly into astrocytes, because we already saw this in our previous experiments. However, graft survival and the differentiation pattern (see later) were not affected by magnetic labeling, and we did not observe any difference between labeled and unlabeled cells, which were detected by staining for MTCO2 or HuNu.

### Morphometric evaluation of the spared white and gray matter

**Figure 3:**
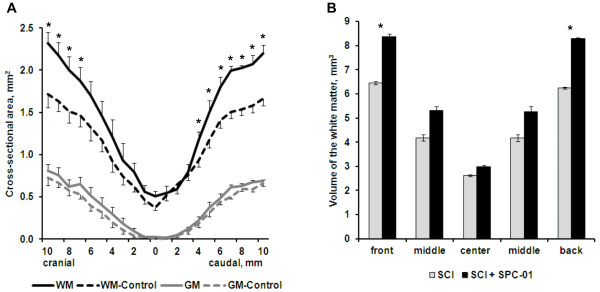
**Morphometric evaluation of the spared white and gray matter.** The white matter (WM) was significantly spared in the spinal cord of grafted animals compared with SCI-only animals (*P < 0.05). No changes were found in the gray matter (GM) (**A**). This was confirmed by measuring the volume (mm^3^) of the white matter in five different parts of the spinal cord (front, 7 to 10 mm cranial; middle front, 3 to 6 mm cranial; center, between 2 mm cranial and caudal to the injury epicenter; middle back, 3 to 6 mm caudal; back, 7 to -10 mm caudal) (**B**).

The area of the white and gray matter was calculated between 1-cm cranial and 1-cm caudal to the injury epicenter. The cross-sectional areas (mm^2^) were plotted at 1-mm increments from the injury epicenter, which was recognized as the smallest area of the spinal cord (Figure 3A). The area of the white matter was calculated by subtracting the areas of the gray matter and the cavity that was created after SCI from the total area of the spinal cord. In grafted animals, when compared with lesioned controls, the white matter was significantly spared in the front segment rostral to 7-mm cranial to the injury epicenter and in the rear segment behind 4-mm caudal to the injury epicenter (*P* < 0.05). No significant difference was noted in the extent of gray-matter sparing (*P* > 0.05). This was clearly shown when the volume (mm^3^) of the white matter was compared in five different parts of the spinal cord (front, 7 to 10 mm cranial; middle front, 3 to 6 mm cranial; center, between 2 mm cranial and caudal to the injury epicenter; middle back, 3 to 6 mm caudal; back, 7 to 10 mm caudal) (Figure 3B). No statistically significant differences were found around the injury epicenter (*P* > 0.05). The sparing of the white matter was prominent throughout the spinal cord, apart from the injury epicenter and the transplanted area.

### *In vivo* cell survival and differentiation

**Figure 4:**
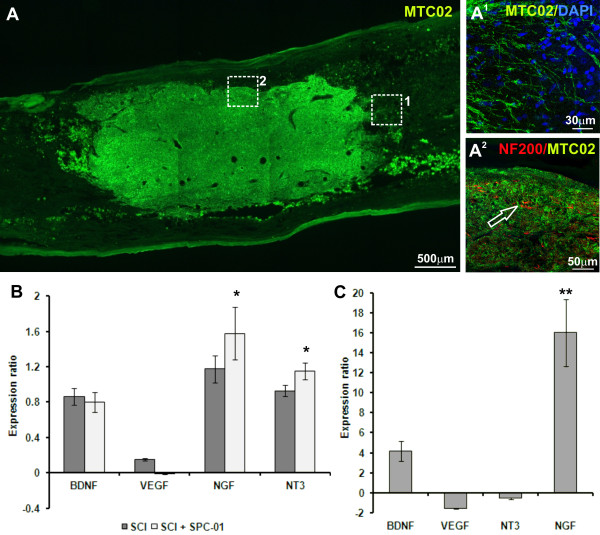
**The survival and trophic effect of SPC-01 cells 8 weeks after transplantation.** The robust engraftment (MTCO2, green) of SPC-01 cells in a lesioned rat spinal cord was observed 8 weeks after transplantation (**A**). At the site of contact with the intact tissue, SPC-01 cells migrated out of the graft (**A**^**1**^, higher magnification of **A**). The processes of only a few host cells entered the implant (**A**^**2**^). qPCR analysis revealed that the level of expression of the rat genes *Ngf* and *Nt-3* was increased in the spinal cords of both control and grafted rats compared with healthy rats; the increase was significant only in the spinal cord tissue of animals grafted with SPC-01 cells (**B**) (*P < 0.05). (**C)** Changes in the expression of the human genes *BDNF, VEGF, NT3,* and *NGF* in SPC-01 cells 8 weeks after transplantation into a lesioned rat spinal cord, compared with the same cells before transplantation (**P < 0.005).

SPC-01 cells were tested in the lesioned rat spinal cord in terms of their survival and differentiation into neuronal and glial phenotypes. In total, 5 × 10^5^ cells were implanted into the center of a spinal cord lesion. The engrafted cells were identified either by GFP positivity or by immunohistochemical staining for the human-specific markers HuNu or MTCO2. Because the majority of the grafted cells lost their GFP positivity 8 weeks after transplantation, we therefore had to rely on staining for human-specific markers (Figure 4A). The animals were killed 8 or 17 weeks after transplantation, and cell survival was evaluated by examining serial longitudinal sections of the lesioned spinal cord at both time points. The results showed that the cells robustly survived in the lesion: during the entire experiment, we found grafts in 19 of 20 animals. Grafted cells also did not form tumors during the entire period of observation, and 8 weeks after transplantation, the Ki67 index was 3.24% ± 0.12%. The cells formed several “densely packed clouds” and filled the lesioned tissue over a few millimeters along the longitudinal axis of the spinal cord (Figure 4A), creating their own microenvironment with a few host cells. When the cells came into close contact with the uninjured spinal cord tissue, they grew into the host tissue: some cell bodies spread out of the cell mass into the host tissue and extended their processes (Figure 4A^1^).

Quantitative analyses of the survival of SPC-01 cells were made from serial cross sections. The total volume of the cell mass remaining 8 weeks after transplantation was 0.29 mm^3^ ± 0.06 mm^3^ on average, and the percentage of surviving SPC-01 cells was 17.4% ± 2.7%.

Eight weeks after transplantation, grafted cells expressed early neural markers such as NSE and nestin, the early oligodendroglial marker Olig2, and the astroglial marker GFAP (all data shown in Additional file 4: Figure S2). Staining for NF200 (heavy-chain neurofilaments) showed the ingrowth of neurofilaments from the host tissue into the “cloud” of transplanted cells (Figure 4A^2^).

To evaluate the effect of transplanted SPC-01 cells on endogenous regenerative processes, we examined the gene expression of several neurotrophic factors: *Bdnf*, *Vegf, Ngf,* and *Nt3,* by using qPCR (Figure 4B). Eight weeks after transplantation, the expression of the *Bdnf, Ngf,* and *Nt3* genes was increased in the spinal cords of both control and grafted rats, compared with intact animals. However, only the changes in *Ngf* and *Nt3* gene expression in spinal cord tissue transplanted with SPC-01 cells were significant (*P* < 0.05). qPCR analysis of the expression of the same human genes in SPC-01 cells after their transplantation into the spinal cord revealed the upregulation of *BDNF* and *NGF* and the downregulation of *VEGF* and *NT3* expression compared with the mRNA levels before transplantation (that is, cells in culture; Figure 4C). However, only the change in the expression of *NGF* was significant (*P* = 0.001). It is interesting to note that *NGF* was expressed in transplanted SPC-01 cells at a high level, although the expression of this gene was not detected in the same cells before transplantation.

**Figure 5:**
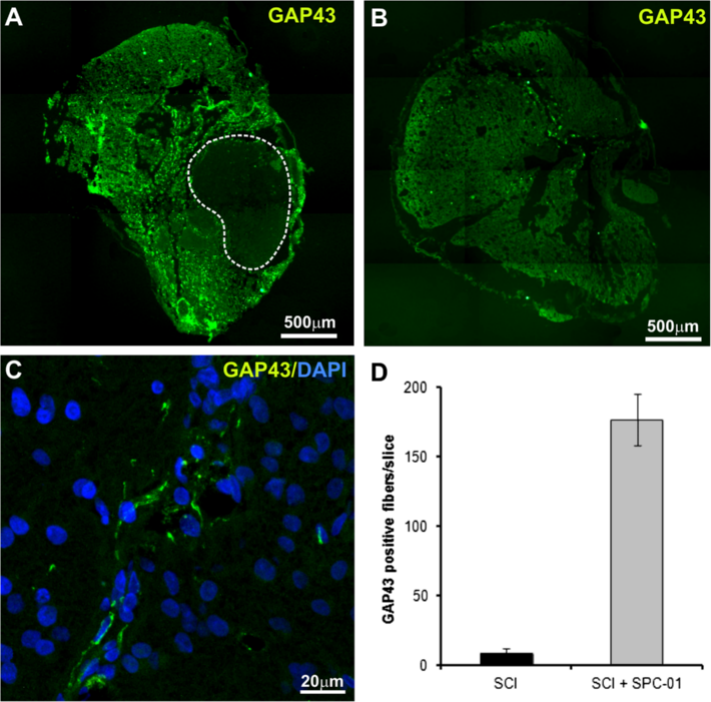
**Axonal sprouting in the injured spinal cords of control rats and rats transplanted with SPC-01 cells.** Staining for GAP43 demonstrates more-intensive sprouting in the injured spinal cord tissue of rats transplanted with SPC-01 cells (**A**) compared with control animals (**B**). (**C)** Higher-magnification view of the transplant area, marked by the white line in (**A)**. (**D)** Quantitative analysis of the number of GAP43-positive fibers in the spinal cord tissue of control and transplanted animals.

Axonal sprouting in the lesion was expressed as the number of GAP43^+^ fibers (Figure 5). Staining for GAP43 was more intense in cross-sections of the spinal cord tissue of transplanted rats (Figure 5A) than in sections from control animals (Figure 5B). The number of GAP43^+^ axonal fibers per section was significantly increased in grafted animals (176.55 ± 18.26) compared with control rats (8.41 ± 3.35) (Figure 5D). The grafted cells were not GAP43^+^, indicating that all sprouting came from endogenous host axons.

**Figure 6:**
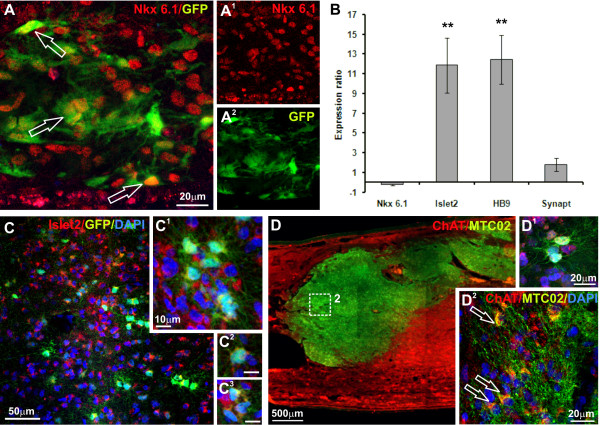
**Differentiation of transplanted SPC-01 cells toward a motor neuronal phenotype.** The expression of motor neuron-specific markers in SPC-01 cells at 8 and 17 weeks after transplantation. Eight weeks after transplantation, 25.31% ± 2.15% of SPC-01 cells were positive for Nkx6.1 (**A**, **A**^**1**^, **A**^**2**^). At the same time, according to the results of qPCR analysis, the expression of the *Nkx6.1* gene in SPC-01 cells did not change, compared with the level of expression seen in the cells before transplantation. However, the expression of the more advanced motor neuron genes *Islet2* and *HB9* in transplanted cells significantly increased (**P < 0.005) compared with that observed in SPC-01 cells before transplantation (**B**). Seventeen weeks after transplantation, SPC-01 cells were positive for Islet2 (**C**; **C**^**1**^, **C**^**2**^, **C**^**3**^: higher magnification of **C**) and ChAT (**D** and **D**^**2**^: higher magnification of **D**; **D**^**1**^: staining for ChAT in combination with GFP and DAPI).

To examine the ability of SPC-01 cells to differentiate toward motor neurons after transplantation into the injured rat spinal cord, we investigated the expression of motor neuron-specific markers. Immunohistochemical staining revealed that 8 weeks after transplantation, 25.31% ± 2.15% of SPC-01 cells were positive for Nkx6.1 (Figure 6A), whereas qPCR analysis did not reveal changes in the expression of this marker compared with the levels seen in SPC-01 cells before transplantation (Figure 6B). However, a highly significant (*P* < 0.005) upregulation in the expression of *Islet2* and *HB9* in transplanted SPC-01 cells was detected with qPCR at the same time point. Eighteen weeks (4 months) after SCI, Islet2 expression in transplanted SPC-01 cells was also detected immunohistochemically (Figure 6C), as well as the expression of choline acetyltransferase (ChAT) (Figure 6D). Orthogonal projections for Figures 6A, C, and D are presented in Additional file 5: Figure S3. These results suggest that a certain population of SPC-01 cells is able to differentiate toward a motor neuron phenotype after transplantation into the injured rat spinal cord. We speculate that this population consists of approximately 25% of the total number of transplanted cells.

**Figure 7:**
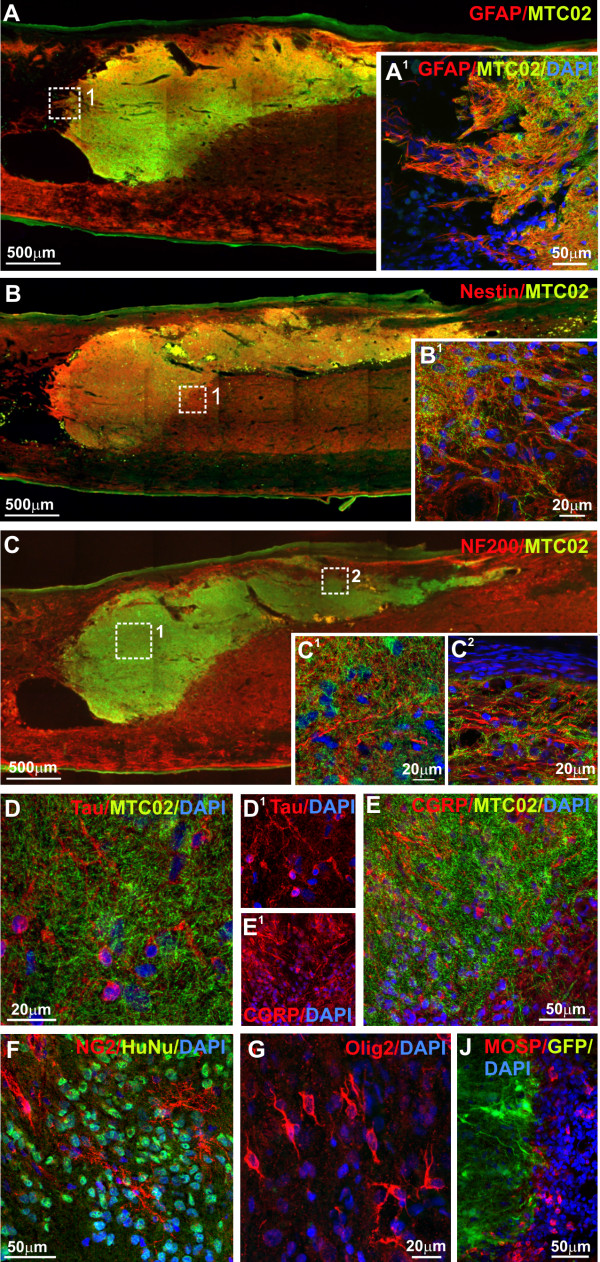
**The incorporation of host tissue elements into the graft 17 weeks after transplantation.** SPC-01 cell survival and migration 17 weeks after transplantation was similar to that seen at 8 weeks. Transplanted cells were positive for GFAP (**A** and **A**^**1**^: higher magnification of **A**) and nestin (**B** and **B**^**1**^: higher magnification of **B**). At the same time, the grafts were infiltrated by endogenous cells and cell elements positive for NF200 (**C** and **C**^**1**^ and **C**^**2**^: higher magnification of **C**), Tau (**D** and **D**^**1**^), CGRP (**E** and **E**^**1**^), and NG2 (**F**), which suggests good communication with the host tissue. In the host nervous tissue adjacent to the transplants, endogenous cells positive for Olig2 (**G**) and MOSP (**J**) were found surrounding the implant.

Seventeen weeks (4 months) after grafting, transplanted SPC-01 cells were less positive for GFAP (Figure 7A) and nestin (Figure 7B) than 8 weeks after grafting, and the expression was confined to individual fibers. However, at this time, the grafted cells were not positive for Olig2, as was the case 8 weeks after transplantation, and were positive for CNPase (Additional file 4: Figure S2F), which suggests their differentiation toward an oligodendroglial phenotype. Co-staining for NF200 (Figure 7C), Tau (Figure 7D), CGRP (Figure 7E), and NG2 (Figure 7F) together with markers of human cells (MTCO2 or HuNu), revealed that newly forming host tissue elements are intensively incorporating into the graft and communicating with grafted cells, because the staining for endogenous tissue elements and the staining for human markers did not colocalize. In the host nervous tissue adjacent to the transplants, endogenous cells positive for Olig2 (Figure 7G) and MOSP (Figure 7J) were found.

## Discussion

In the present study, we used clone 01 of human fetal neural stem cells (SPC-01) derived from fetal spinal cord as a cell source to treat SCI in adult rats. We demonstrate that SPC-01 cells differentiated toward motor neuronal, astroglial, and oligodendroglial phenotypes in the injured spinal cord and promoted functional recovery. Generally, the transplantation of different types of neural stem cells often results in functional improvement and to some extent in neural differentiation as well [13-15,30]. However, SPC-01 cells possess some unique features that make them potential candidates for future clinical use.

Parr and colleagues [31] reported that NSCs derived from adult spinal cord differentiated into astrocytes (31.2%), oligodendrocytes (50.3%), and neurons (less than 1%) after transplantation into the rat spinal cord and that no locomotor improvement occurred [31]. The survival of adult NSCs was very poor, less than 5%. In our study, a single injection of half a million cells into the spinal cord resulted in a large number of surviving cells and facilitated host tissue regeneration 8 weeks after transplantation. This excellent cell survival contributed to the partial restoration of locomotor function (we used only the BBB test and the beam-walking test, because the majority of tests, such as grid walking or rotarod, require weight support and stepping, which were not achieved by the control animals). Morphometric measurements revealed that the white matter was markedly spared at sites remote from the injury epicenter (that is, not around the injury epicenter where the transplants were seeded). The total volume of spared white matter in a 21-mm-long spinal cord segment was 22% greater in the transplanted group compared with that in the control group. A single injection of a large number of cells enabled the extremely good survival of the transplanted cells in the lesioned area, as confirmed by MR images, Prussian blue staining, and staining for the human mitochondrial marker MTCO2. The cell-survival rate of 17% is particularly noteworthy, considering that the cell-survival rate was less than 5% in the published work of Parr and colleagues [31]. In addition, even the injection of a rather large volume of cells did not result in any hyperproliferation or tumor formation. The reason for the better survival can be either the triple immunosuppression and/or the injection of a large cell bolus (5 × 10^5^/5 μl), which may help the transplanted cells to create a more-permissive environment in the hostile lesion, because three injections of fewer cells (1 × 10^5^/1 μl) resulted in worse cell survival.

To enable *in vivo* detection of the graft, cells were labeled before transplantation with PLL-SPIO nanoparticles. Several studies have used magnetically labeled cells to monitor the fate of transplanted cells within the organism [22,32,33]. Cells labeled with iron-oxide nanoparticles (at a similar concentration) can migrate, differentiate, and improve functional outcome in different diseases [11,34]. It is possible to monitor SPIO-labeled neural stem cells for 18 weeks after transplantation [35]. The cells can differentiate into neuronal and glial lineages and have neuronal-like electrophysiological characteristics, with no signs of tumor formation. Similarly, subventricular zone (SVZ) cells labeled by ferromagnetic particles were transplanted intracisternally into a rat model of stroke. The transplanted cells selectively migrated toward the ischemic parenchyma, and the grafted animals exhibited significant improvement of their neurologic function [36]. In our experiments, we followed the animals by using MRI only for 8 weeks. The graft was clearly visible at the cranial side of the lesion, although the labeling efficiency was only 57%. By 8 weeks after transplantation, the transplanted cells were mainly nestin- and GFAP-positive, no matter whether the cells were nanoparticle-labeled or not. Also, no difference was observed in behavioral tests between animals that were transplanted with labeled or unlabeled cells. However, the morphometric evaluation of the spared white and gray matter, as well as the qPCR analyses, were performed in animals grafted with unlabeled cells, although it was shown that the secretion profile of ferumoxide-labeled human bone marrow, mesenchymal cells was not impaired by the labeling, including the production of growth factors and cytokines mediating the recovery effect [37]. To study more precisely the effect of SPIO nanoparticles on *in vivo* differentiation and the dynamics of nanoparticle expulsion, further long-term studies would have to be performed.

We have seen functional improvement as early as 3 weeks after transplantation. However, 5 weeks later (that is, 8 weeks after transplantation), grafted SPC-01 cells still expressed mainly progenitor markers, such as Olig2, nestin, and NSE, and were GFAP positive. Conversely, at this same time, the robust sprouting of endogenous neurons, positive for GAP43, was observed, most likely due to a paracrine effect of the graft by which the expression of the *NGF* gene was significantly upregulated. Moreover, the transplantation of SPC-01 cells led to the upregulated expression of the rat neurotrophin genes *Nt3* and *Ngf* compared with lesioned control animals. Neurotrophic factors such as NGF, BDNF, and NT3 play critical roles in axonal growth and in the survival of existing neurons [38-41]. Consistent with these reports, we observed that SPC-01 cell transplantation promoted functional recovery after SCI. Therefore, we suggest that the early significant improvement in motor and sensory tests was mediated by the increased expression of neurotrophic factors by the transplanted SPC-01 cells, especially NGF. Human cells, although they are transplanted into rodent tissue, mature much more slowly than their mouse or rat counterparts. A similar effect was observed in our study when NSCs derived from human iPS cells were injected into animals with middle cerebral artery occlusion (a model of stroke). Functional outcome and the protection of the host substantia nigra from atrophy were also observed much earlier than the slow maturation of human GABAergic neurons and their innervation of the host substantia nigra [42]. Here, we demonstrate that around 25% of the transplanted SPC-01 cells were able to differentiate toward a motor neuronal phenotype. At 8 weeks after transplantation, increased expression levels of motor neuron-specific markers were detected with qPCR; however, only the early motor neuron marker Nkx6.1 was detected immunohistochemically. Nevertheless, 2 months is too short a period for the terminal differentiation and maturation of human cells *in vivo*. It was 2 months later (week 17) when we were able to detect immunohistochemically Islet2 and ChAT, markers of postmitotic motor neurons, in grafted SPC-01 cells. We did not study the incorporation and communication of newly formed motor neurons with the host tissue. This aspect of the stem cell therapy effect on recovery after SCI remains as a task for future experiments.

Neuronal differentiation does not equal neural tissue reconstruction. However, during our experiments, we observed the progressive ingrowth of host axons into the implant. Seven weeks after grafting, the SPC-01 cells formed dense clouds, and only sparse host neurofilaments were detected growing into the graft. Two months later, the graft was robustly grown through with neurofilaments, and we found host axons in the graft center. Apart from neuronal communication, we observed clusters of NG2- Olig2-, and MOSP-positive cells surrounding the SPC-01 implants. It was shown that oligodendrocytes possess neurotrophin receptors [43], and we can speculate that host oligodendrocytes and their progenitors are attracted by the elevated concentration of neurotrophic growth factors in the graft.

## Conclusions

The SPC-01 cell line can be expanded in large quantities and, when implanted into an animal model of SCI, the cells robustly survive in the lesion, express neurotrophins, stimulate the expression of host neurotrophic genes, and facilitate the sprouting of endogenous GAP43-positive axons. All of these actions lead to improvements in locomotor and sensory functions and to the sparing of the white matter in the short term. In the long term, about 25% of transplanted SPC-01 cells can slowly mature into motor neurons and participate to some extent in tissue reconstruction. SPC-01 cells were derived by using the same protocol as the CTX0E03 immortalized cell line derived from human cortical neuroepithelium [18]. These cells are currently undergoing testing in a human clinical trial for stroke patients. Similarly, our results represent a proof-of-concept that conditionally immortalized neural stem cell lines from human spinal cord could be used in the future for cell therapy in SCI patients.

## Abbreviations

4-OHT: 4-Hydroxy-tamoxifen; A1ctb: actin beta; BBB test: Basso, Beattie, and Bresnahan test; BDNF: brain-derived neurotrophic factor; CGRP: calcitonin gene-related peptide; ChAT: choline acetyltransferase; CNPase: 2ʹ,3ʹ-cyclic nucleotide 3ʹ-phosphodiesterase; Cq: cycle of quantification; DMEM:F12: Dulbecco modified Eagle medium, nutrient mixture F-12; DNA: desoxyribonucleic acid; EGF: epidermal growth factor; FGF: fibroblast growth factor; GABA: γ-aminobutyric acid; GAP43: growth-associated protein 43; GAPDH: glyceraldehyde 3-phosphate dehydrogenase; GFAP: glial fibrillary acidic protein; GFP: green fluorescent protein; HB9: homeobox 9; HBSS: Hank balanced salt solution; HuNu: human nuclei; iPS cells: induced pluripotent stem cells; Islet2: -insulin-related protein 2; MOSP: myelin/oligodendrocyte-specific protein; MRI: magnetic resonance imaging; mRNA: messenger ribonucleic acid; NF160: neurofilament 160; NF200: neurofilament 200; NG2: chondroitin sulfate proteoglycan; NGF: neural growth factor; NSCs: neural stem and progenitor cells; NSE: neurospecific enolase; NT3: neurotrophin 3; PFA: paraformaldehyde; PLL-SPIO: poly-L-lysine-coated superparamagnetic-iron-oxide; qPCR: quantitative polymerase chain reaction; REST: relative expression software tool; RNA: ribonucleic acid; SCI: spinal cord injury; SEM: standard error of the mean; SPC: spinal precursor cell; SVZ: subventricular zone; UCOE: ubiquitous chromatin opening element; VEGFA: vascular endothelial growth factor A.

## Competing interests

Prof. Jack Price is a consultant for ReNeuron PLC; other authors have nothing to declare.

## Authors’ contributions

TA and NR were responsible for the conception and design of the experiments, the collection and/or assembly of the data, data analysis and interpretation, and manuscript writing. VH, KT, PP, and MK were involved in data collection, analysis, and interpretation. GC provided study material and was involved in manuscript writing. JP was involved in the conception and design of the experiments and provided study material and administrative support. PJ and ES were involved in the conception and design of the experiments, data analysis and interpretation, and manuscript writing; they also provided financial and administrative support. All of the authors read and approved the final manuscript for publication.

## Supplementary Material

Additional file 1: Table S1 The numbers of animals used in all parts of the study. Click here for file

Additional file 2 **Description of the methods for the determination of cell viability, labeling efficiency, and fluorescence-activated cell-sorting analysis.** Description of the results of SPC-01 cells FACS analysis. Click here for file

Additional file 3: Figure S1 Fluorescence-activated cell-sorting profiles of pluripotent and neural markers in SPC-01 human fetal neural stem cells. Click here for file

Additional file 4: Figure S2 Expression of early neural and glial markers by SPC-01 cells 8 weeks after transplantation into SCI. Eight weeks after transplantation, SPC-01 cells expressed the early neural markers NSE (S2A and B) and nestin (S2C), the early oligodendroglial marker Olig2 (S2D), and the astroglial marker GFAP (S2E). Seventeen weeks after transplantation, SPC-01 cells were positive for CNPase (S2F). Click here for file

Additional file 5: Figure S3 Orthogonal projection for Figure 6 images. Click here for file
