# Supplementary material for: Human conditionally immortalized neural stem cells improve locomotor function after spinal cord injury in the rat
Source: Stem Cell Res Ther. 2013 Jun 7;4(3):68. doi: 10.1186/scrt219 (PMC3706805; doi:10.1186/scrt219)
Supplement: Additional file 1: Table S1 — The numbers of animals used in all parts of the study. [file scrt219-S1.pdf]

Table 1: The numbers of animals used in all parts of the study.

	SCI only	SCI + SPC-01
8weeks		
Behavioral tests	23 (4 of them were used as controls for MRI)	28 (4 of them were used for MRI and 20 for IHC)
PCR*	4	4
GAP43 cross-sections	8	7
17 weeks		
IHC	-	5

*- for PCR 4 healthy animals (without SCI) were used as intact controls.
