# Supplementary material for: Human conditionally immortalized neural stem cells improve locomotor function after spinal cord injury in the rat
Source: Stem Cell Res Ther. 2013 Jun 7;4(3):68. doi: 10.1186/scrt219 (PMC3706805; doi:10.1186/scrt219)
Supplement: Additional file 2 — Description of the methods for the determination of cell viability, labeling efficiency, and fluorescence-activated cell-sorting analysis. Description of the results of SPC-01 cells FACS analysis. [file scrt219-S2.pdf]

Methods

Determination of cell viability

The viability of PLL-SPIO labeled cells was determined using a colorimetric assay based on the cleavage of the tetrazolium salt WST-1 (4-[3-(4-Iodophenyl)-2-(4-nitrophenyl)-2H-5-tetrazolio]-1,3-benzene disulfonate) (Roche Diagnostics, Mannheim, Germany) to a highly water-soluble formazan dye by mitochondrial dehydrogenases in viable cells [1]. SPC-01 cells were plated on 96-well plates (TPP, Trasadingen, Switzerland) at a density of 2×10^3 cells/well. The cells were cultured and labeled with iron oxide nanoparticles as described above. On the day that the nanoparticles were withdrawn (day 3), 10 μl of the WST-1 solution was added to 100 μl of culture medium per well, and the cells were kept in an incubator (37°C) for an additional 2 hours. The absorbance was measured using an ELISA plate reader (Tecan Spectra, Tecan Trading, Switzerland) at a wavelength of 450 nm.

Labeling efficiency

Labeling efficiency was determined by manually counting the number of Prussian Blue-stained and unstained cells in five optical fields from each plate using an Axioplan® Imaging II microscope at 100× magnification and a 10×/0.75 objective lens, an AxioCam® digital camera, and AxioVision® 4 software (Carl Zeiss AG, Oberkochen, Germany). The scanned images with manually labeled cells were processed by the MATLAB® 6.0 Image Processing Toolbox™ (MathWorks, Natick, MA), and the presence or absence of a label inside the cells was expressed as the percentage of labeled cells.

Fluorescence-activated cell sorting analysis

SPC-01 cells were dissociated from monolayers by TrypZean (Lonza) for 2-5 min. After rinsing with PBS, the cell suspension (10×10^6 cells/ml and no less than 3×10^5 cells per sample) was used for flow cytometric analysis using a Becton Dickinson FACSAria flow cytometer (BD Bioscience, San Diego, CA). Data analysis was performed using BD FACSDiVa software. To analyze the human neural stem cells, conjugated antibodies against CD29, CD271 (NGFR), HLA-ABC, CD34, CD44 (BD Pharmingen, San Diego, CA), CD15 (SSEA-1), CD56 (NCAM), CD24, CD105 (Exbio Antibodies, Prague, CR), CD133/1, CD133/2, PSA-NCAM (Miltenyi Biotec, Bergisch Gladbach, Germany), nanog, TRA-1-60, CD90, CD184 and SSEA-4 (eBioscience, San Diego, CA) were used, along with unconjugated primary antibodies against nestin and NF70 (abcam, Cambridge, UK), sox2 and βIII-tubulin (BD Pharmingen, San Diego, CA), oct3/4 (R&D Systems, Minneapolis, MN), A2B5 (Miltenyi Biotec, Bergisch Gladbach, Germany) and a secondary rat anti-mouse IgM conjugated with FITC (eBioscience). As negative controls, IgG1 isotype conjugated with FITC or RPE and IgG2a isotype conjugated with RPE (Dako Cytomation, Glostrup, Denmark) were used, as well as rat anti-mouse IgM conjugated with FITC without the addition of the primary antibody.

Results

FACS analysis of SPC-01 cells

To characterize the expression of pluripotent and neural markers in human fetal neural stem cells from the SPC-01 line, a series of FACS analyses was performed prior to transplantation (Fig. S3). The flow cytometry results revealed that SPC-01 cells are negative for TRA-1-60, nanog and SSEA-4 and that only one pluripotent marker, oct3/4, is expressed in 50%

of all cells (48.18±2.59). SPC-01 cells also displayed a moderate to high level (greater than 40% positive cells) of expression of several neuroectodermal and neural markers: sox2, nestin, CD56 (NCAM), A2B5, βIII-tubulin, NF70, CD15, CD24, CD29, CD90, CD133, CD184 and CD271. They also had a surprisingly high level of expression of HLA-I antigen and CD44 – almost 100% – and a low level of expression of HLA-II antigen and CD34.

Taken together, these data confirm that SPC-01 cells display a clear neural profile in terms of their marker expression. At the same time, these cells are negative for pluripotent markers, which suggests that they are safe for use in transplantation experiments.

References

1. Ishiyama T, Watanabe K, Akimoto Y, Ueno H, Yamada K, Koike M, Hino K, Tomoyasu S, Tsuruoka N, Ota H: **Circulating abnormal cells detected in a patient with immunoblastic lymphadenopathy.** *Intern Med* 1993, **32:**455-458.
