# Supplementary figures and images for: Human conditionally immortalized neural stem cells improve locomotor function after spinal cord injury in the rat
Source: Stem Cell Res Ther. 2013 Jun 7;4(3):68. doi: 10.1186/scrt219 (PMC3706805; doi:10.1186/scrt219)

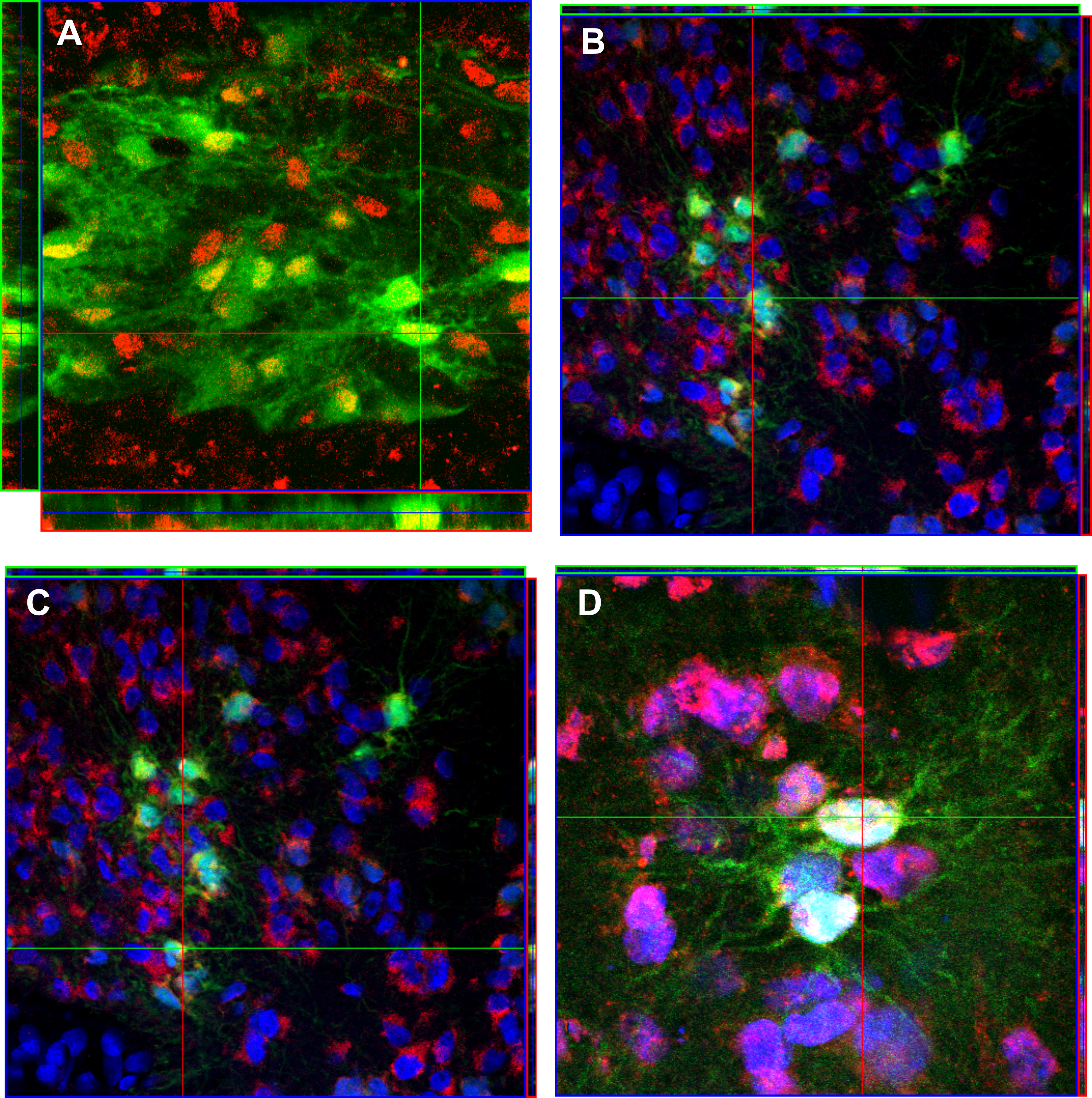

Supplement: Additional file 3: Figure S1 — Fluorescence-activated cell-sorting profiles of pluripotent and neural markers in SPC-01 human fetal neural stem cells. [file scrt219-S3.tiff]

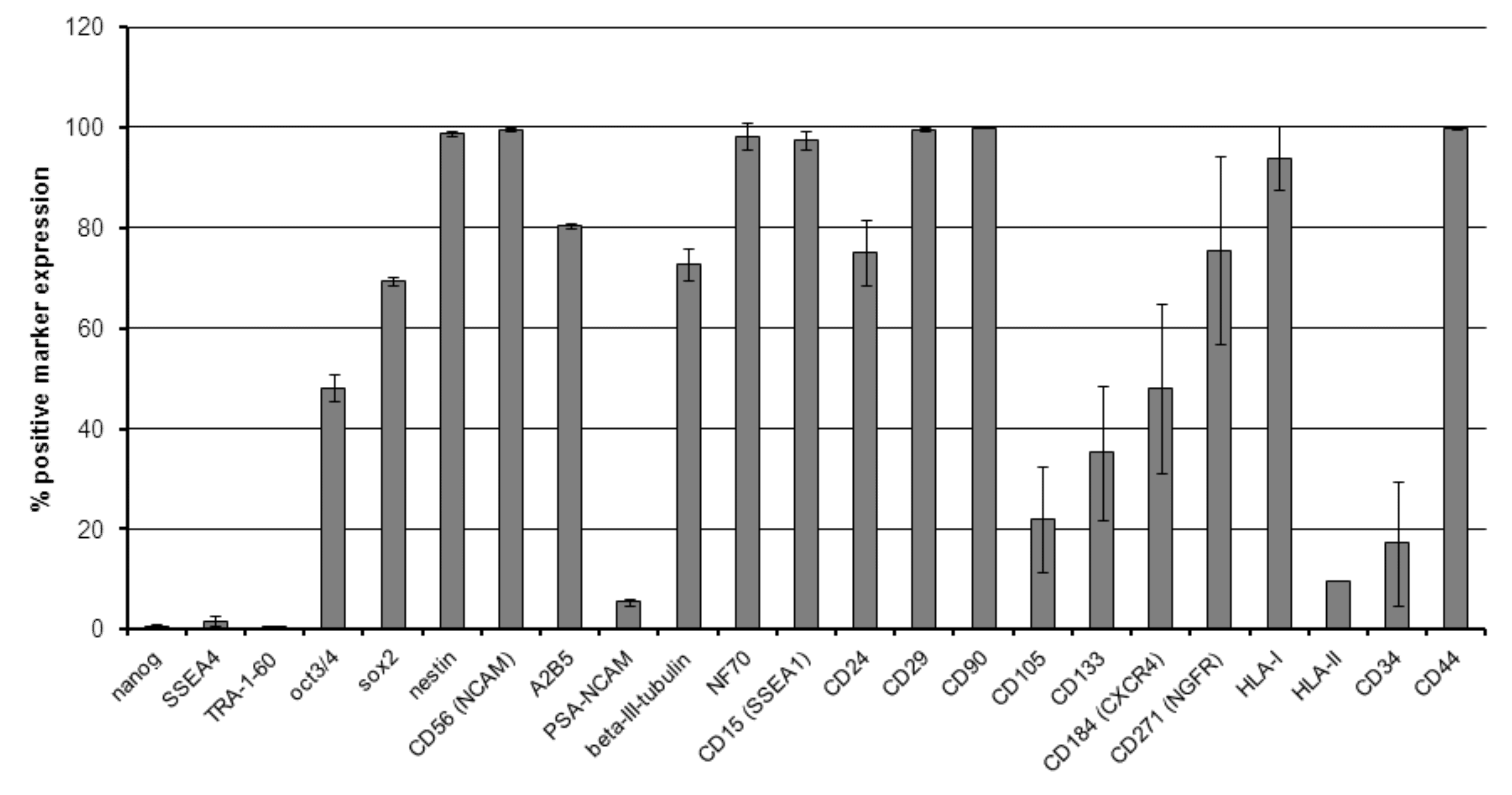

Supplement: Additional file 4: Figure S2 — Expression of early neural and glial markers by SPC-01 cells 8 weeks after transplantation into SCI. Eight weeks after transplantation, SPC-01 cells expressed the early neural markers NSE (S2A and B) and nestin (S2C), the early oligodendroglial marker Olig2 (S2D), and the astroglial marker GFAP (S2E). Seventeen weeks after transplantation, SPC-01 cells were positive for CNPase (S2F). [file scrt219-S4.tiff]

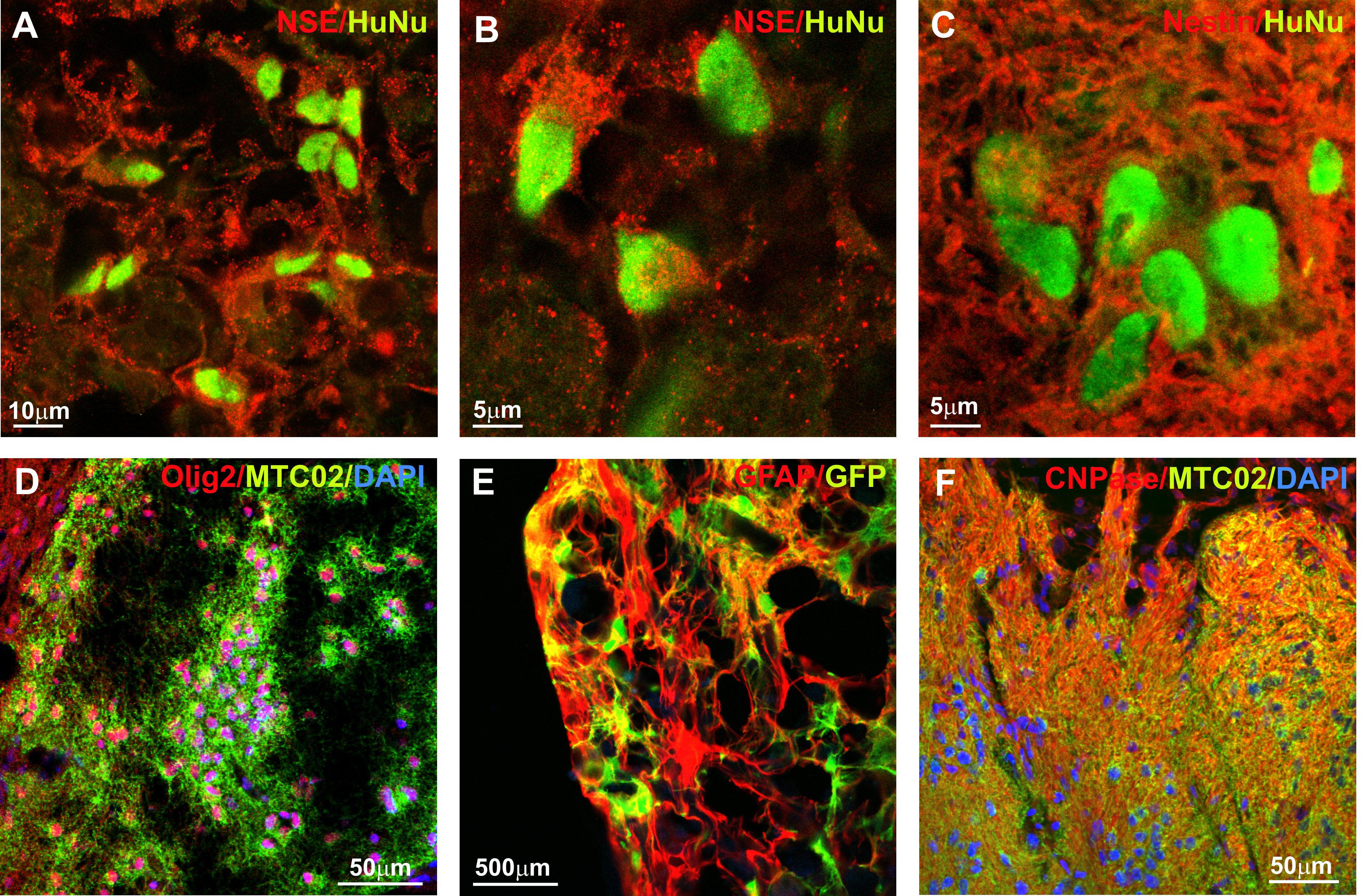

Supplement: Additional file 5: Figure S3 — Orthogonal projection for Figure 6 images. [file scrt219-S5.tiff]
